# The *mBio* American Academy of Microbiology Submission Track in 2017

**DOI:** 10.1128/mBio.00050-17

**Published:** 2017-02-07

**Authors:** Arturo Casadevall, Thomas Shenk

**Affiliations:** a Department of Molecular Microbiology and Immunology, Johns Hopkins Bloomberg School of Public Health, Baltimore, Maryland, USA; b Department of Molecular Biology, Princeton University, Princeton, New Jersey, USA

## EDITORIAL

When *mBio* was launched in 2009, it offered two tracks for submission of manuscripts. The regular track handles manuscripts submitted to *mBio* in a conventional manner, which involves an initial evaluation by editors and subsequent blind peer review for those manuscripts found suitable for the journal. The second track, known as the American Academy of Microbiology (AAM) track, involves the submission of manuscripts by members of the AAM accompanied by author-solicited reviews after the paper has been revised according to the comments and criticisms of the reviewers. The reviewers are also asked to attest that they consider the manuscript to be in the top 10% of the science in the field. AAM members are limited to one AAM track submission per year. The mechanics of the AAM track were described recently (1). It has worked well over the past 8 years; we have no evidence that it has been abused, and AAM papers are cited with the same frequency as regular papers (1). In 2017, *mBio* will make two changes to the process by which AAM track manuscripts are handled.

The first change is that all AAM contributions will now be subject to accept- or reject-only decisions. In the past, editors had the choice of sending the paper out for a blind review, but this option defeated the purpose of the AAM track, which was to provide a mechanism for rapid acceptance. Experience has shown us that, when editors felt that an additional blind review was needed, there was a high likelihood that the paper would ultimately be rejected. Presumably, this reflected the fact that the editor detected something amiss with the manuscript and/or the reviews, which was then confirmed by the additional review. Furthermore, this change in policy harmonizes the procedures of *mBio* with those of *mSphere*, which has recently launched mSphereDirect, a submission track using author-initiated reviews, which are then subjected to only accept or reject decisions (2). We note that mSphereDirect has instituted stringent and detailed criteria for who may be a reviewer in author-initiated reviews (http://msphere.asm.org/content/mspheredirect-instructions-authors#SEmSD), which *mBio* will look into adopting as well.

The second change is to ask authors using the AAM track to return the paper to the reviewers after revision for their approval. Although we recognize that this will add more friction to the process, we feel that this step will provide an additional quality control measure that might improve the final product. This change in policy is also designed to protect the reviewers, since their names are published with the article, and it seems fair that they have approved the final draft. mSphereDirect has already instituted this step as part of the author-initiated review track (2), and adopting the same practice at *mBio* again harmonizes the procedures followed by the two ASM journals.

One of the concerns when the AAM track was designed was that authors would deliberately seek easier reviewers and that reviewers would in turn be less likely to be critical given that they would be giving their criticisms directly to individuals who knew them. In fact, this concern was the subject of recent correspondence to *mBio* by one of our editors (3). We feel that publishing the reviewer names with the *mBio* paper provides an additional quality control step that also addresses this concern, since scientists in the field who read an AAM track paper will know both the authors and the reviewers and they will be able to rapidly judge the appropriateness of the individuals who reviewed the paper. This openness should also encourage authors to seek the best reviewers for their paper, since the stature of the reviewers listed will then reflect on the publication. Ultimately, the reputations of both reviewers and authors are on the line during the author-initiated review process, and we believe that, given the importance of reputation to how scientists are perceived, this openness provides an important check on any attempt to game the process.

Many of the AAM track manuscripts that are declined are rejected because the editor did not think that the work was in the top 10% of the field or doubted its importance. Although these assessments are always a matter of judgment, we feel that our editors can instinctively judge the importance of a paper. *mBio* editorial policies encourage editors to reject AAM track papers if there is any question about the importance of the study, the quality of the reviews, and/or the author’s response to the reviewers. Editors are shielded from their decisions, since all rejection letters are sent under the signature of the editor in chief.

Given the success of the AAM track, *mBio* is planning to continue this experiment in publishing while constantly looking for ways to improve it. At a time when there is relatively little experimentation in scientific publishing, we feel that the AAM track provides a mechanism for rapid publication where the authors have more control over the review process. The continuation of the AAM track also maintains the alliance between *mBio* and the AAM, which has been beneficial to both parties, as *mBio* has benefited from outstanding manuscripts and this perk has enhanced the value of election to the AAM, resulting in more candidate nominations (1). Some have argued to the editor in chief that if the AAM track is so successful, it should be extended to all authors. *mSphere* has now created a submission track that provides precisely that option with mSphereDirect (2).

Finally, we comment on what we think are the most appropriate papers for the AAM track. The ideal AAM track submission is a paper describing outstanding work at the top of the field, and the authors are using the track to ensure rapid publication. Papers that challenge dogma and that are likely to encounter flak in entrenched fields are particularly welcomed, provided that they are well done and the conclusions supported by data.
